# Early puberty in short-haired Guinea pigs kept in laboratory animal facilities

**DOI:** 10.1590/1984-3143-AR2021-0068

**Published:** 2022-04-22

**Authors:** André Silva de Matos, Tatiana Kugelmeier, Diva Anelie de Araújo Guimarães, Klena Sarges Marruaz da Silva

**Affiliations:** 1 Instituto de Ciência e Tecnologia em Biomodelos, Fundação Oswaldo Cruz, Rio de Janeiro, RJ, Brasil; 2 Instituto de Ciências Biológicas, Universidade Federal do Pará, Belém, PA, Brasil

**Keywords:** Cavia porcellus, reproduction, vaginal cytology, puberty, estrus detection, laboratory animal science

## Abstract

Lab animals, such as Guinea pigs (*Cavia porcellus*), are crucial for scientific development, as they play an important role in the development and quality control chain of vaccines and drugs distributed by the Brazilian public health system. Investigating their biological and physiological parameters is fundamental to raise and keep these animals, so the handling of the facilities that hold them can be updated whenever new information comes up, with the well-being of the animals and alignment with the 3 Rs in mind. In the search for understanding reproductive aspects of Guinea pigs, the present study had the main goal of studying puberty by means of estrous cycle analysis in short-haired Guinea pigs. Guinea pigs have a vaginal occlusive membrane that covers the vaginal orifice. Its rupture takes place gradually and naturally, moments before labor and during estrus. The present study followed 42 females as for the presentation of the vaginal occlusive membrane. Once the membranes ruptured spontaneously, a swab was collected to study vaginal cytology. Membrane rupture was observed in 39 females; six females showed membrane rupture with less than 21 days of age (17 to 21 days). Twenty-three females were characterized as being in estrus due to cytology showing a prevalence of anucleated superficial cells. One of these females was younger than 21 days old. The opening of the vaginal occlusive membrane took place most frequently in intervals between 17 and 18 days, and the membrane remained open between one and three consecutive days. It was possible to follow three cycles of membrane opening on six females. The present study showed the need to adapt handling guidelines for *C. porcellus* kept in research animal facilities. The early age of puberty imposes the need of separate the female daughters from their fathers at 16 days old.

## Introduction

Guinea pigs (*Cavia porcellus)* are very important in the field of science, especially to the quality control of vaccines and drugs distributed via the Brazilian public health service (Couto, 2002). In addition to providing blood and blood products for the development of diagnostic methods for diseases.

These species are polyestrous and non-seasonal, with spontaneous ovulation and corpus luteum with active secreting activity, with estrous cycle varying between 14 and 16 days (Kühnel and Mendoza, 1992; Wang et al., 2010; Li and Shen, 2015), or 15 and 17 days (Lilley et al., 1997; Hubrecht, 2010).

From the anatomical point of view, these animals have a membrane occluding the vaginal canal. That disappears and regenerates every reproductive cycle. This occurs due to hormonal influence, throughout the life of the females (Stockard and Papanicolaou, 1919). In the estrous cycle, the vaginal canal opens remains open between three and seven days, coincinding with the proestrus, estrus and metaestrus phases (Luna et al., 2003). In mature females, the membrane remains open for one to three days during estrus, but in juvenile females this phase can last longer (Araníbar and Echevarría, 2014). According to Li and Shen (2015), vulvar morphological characteristics in Guinea pigs suffer small alterations in between the different phases of the estrous cycle: swelling, redness, and secretion during proestrus and estrus, and partially closed, light-colored vaginal membrane during the metaestrus.

Pregnancy in Guinea pigs lasts 68 days on average and the development is precocious from embryonic life. The first coat are still observed at this stage, at 50 days of gestation, and at 60 days the coat already presents the characteristic colour throughout the body (Viana et al., 2019). In a study on embryonic development, it was observed that, after 30 days of gestation, it is possible to distinguish males and females, observing the formation of urogenital folds. At 45 days, the folds show characteristics of the future labia and, in the final of the pregnancy, the labia and the vaginal occlusion membrane are formed (Santos et al., 2016).

Guinea pigs are early developing animals. The newborns weigh between 60 and 100 g, with fully haired, ears and eyes open, teeth, eating solid foods and drink water (Hubrecht, 2010). It is known that puberty occurs between 55 and 70 days of age; the first estrus usually takes place at the age of 68+21 days, on average (Andrade et al., 2002; Lapchik et al., 2017). However, females can reach sexual maturity with less than one month of age, with a body mass of 300 g (Kaliste, 2007; Hubrecht, 2010). Corroborating this information, a unique study reported the rare occurrence of estrus at only 20 days of age (Rood and Weir, 1970).

According to guides and manuals to care this species, the weaning should take place around 21 days old (Andrade et al., 2002; Trillmich et al., 2006; Hubrecht, 2010; Lapchik et al., 2017) or when the animals are weighing ≥180 grams (Brasil, 2016). However, if the Guinea pigs present an estrus when they are younger than 21 days, period during which they are still in the family group, this makes it possible for them to be impregnated by their fathers. The risk of providing pregnant animals is an ethical concern and an important experimental variable. In addition, establishing a proper reproductive management helps reduce an excessive number of animals in the facilities.

The premise of the present study was therefore the existance of early puberty in short-haired Guinea pigs, allowing for unplanned pregnancies while the animals remain in their family groups at a research facility. To confirm this hypothesis, females were monitored via vaginal cytology and observation of the vaginal occlusive membrane.

## Methods

### Animal facilities and handling

The study was carried out at the Service of Raising of Rodents and Lagomorphs of the Institute of Science and Technology in Biomodels – ICTB of the Oswaldo Cruz Foundation (Rio de Janeiro, Brazil).

The animal facilities in which the Guinea pigs were held into autoclavable polypropylene cages measuring 90 x 60 x 30 (length x width x height), in family groups with 6 animals (females, breeding males and puppies upon completing 21 days of age).

The environment was kept at temperatures between 19 and 21 °C, relative air humidity between 40 and 60%, and light-dark cycles of 12:12 hours. All food and materials offered to the animals were submitted to chemical and/or physical decontamination processes. Nutritional handling consisted of specific Guinea pig feed (Quimtia®), supplemented with vitamin C (Vetec Quimica Fina LTDA), a multivitamin (Organnact®), and autoclaved water.

### Experiment outline

At 14 days old the puppies received microchips in the dorsal scapular region for identification.

Observation of vaginal membranes began when the animals turned 15 days old. Every day, between 9 and 10 a.m., the animals were physically restrained, weighed, and had their genital area observed, with a macroscopic evaluation of the vaginal occlusive membrane classified in three stages: Partially open (VMPO) – when had opening of the membrane but not enough for a swab to be introduced; Closed (VMC) - when had vaginal canal completely covered by the membrane. From 30 to 60 days of age, the membrane was inspected every two days. When it was seen opened, cytological samples were collected daily, until observed closed. The macroscopic aspects of the genitalia (color of the vagina, presence of mucus or fluids, swellingo f the vulva) were also recorded daily.

### Vaginal cytology

The vaginal material was collected with an extra-fine cylinder-shaped interdental brush, 3 mm of diameter, soft bristles, damp with saline solution and introduced in the vaginal canal, then rotated to gently exfoliate the vaginal epithelium.

Following the collection, the material was smeared on two microscopy slides, which were identified (sample ID) with a combination of a random number and a letter corresponding to the staining technique to be used (A for rapid panoptic and B for Papanicolaou).

The Romanowsky staining technique (panoptic staining) was made by a commercial kit (LABORCLIN). The Papanicolaou (1933) staining also used a commercial kit (RENYLAB) and was performed according to the author, with adaptations (Kugelmeier et al., 2011). Both were carried out at the SCPRIM/ICTB Reproduction Laboratory at ICTB/Fiocruz.

The quantitative evaluation considered the counting of cell types in absolute numbers, for a total of 200 cells, which was performed by optical microscopy (100 and 400 x zoom). After the counting, the percentage of cell types contained in the vaginal epithelium was calculated. According to the classification used by Guimarães et al. (1997), into the following cells: basal, parabasal, intermediate, nucleated superficial, and enucleated superficial. The qualitative evaluation observed the presence of mucus, leukocytes, fungi, bacteria, cleanliness, and quantity of material on the slide. The presence of a finding was qualified according to the follow criteria, and then recorded in the spreadsheet (vaginal material samples collection): Light-observed in few fields; Moderate- observed in some fields; High- Observed in all fields. The macroscopic evaluation of the vulva, of the distribution of cell types and of the material on the slide was used to characterize the phases of the estrous cycle. Characterization of diestrus considered the analysis of slides collected beginning on the fifth day of membrane opening.

### Analysis

For the description of the results, the following criteria were considered: opening of the occlusive membrane, age, time the membrane remained open and phase of the cycle during which the membrane opened. For the vaginal cytology figures, descriptive statistic, average and standard deviation, standard error, and variation of original data were considered. Variance analysis (ANOVA) was used to compare the number of cell types present in the three cycles observed and the T Test used to compare the averages obtained in the first and second cycles, using the Bioestat free software (version 5.0).

The level of significance used to reject H_0_ (hypothesis of nullity) was 5%, that is, lower than 0.05.

### Ethical aspects

This study was approved by the Ethics Committee for Use of Animals - CEUA Fiocruz, under licence number LW-12/15 and the additive term P39/14.4 for the inclusion of the activity “sampling for vaginal cytology”.

Observation was finished when the animals turned 61 days old. The animals’ management general routine was not modified, except for the observations and sample collections.

## Results

Evaluation of estrous cycle between 15 and 60 days of age.

**Figure 1:**
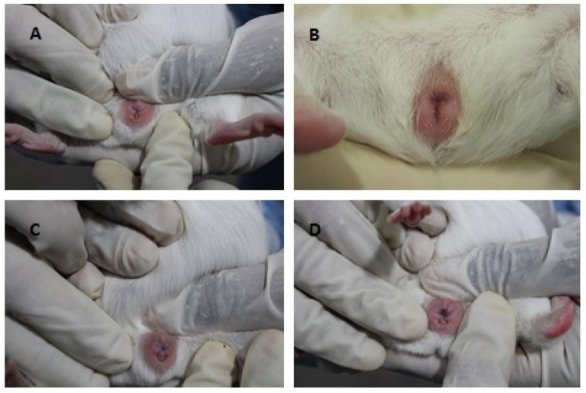
Macroscopic analysis of vaginal occlusive membrane of *Cavia porcellus* between 15 and 60 days old. Vaginal Membrane Closed (A); Swollen vulva and secretion (B); Vaginal Membrane Partially Open (C); Vaginal Membrane Open (D).

The macroscopic evaluation of the vaginal membrane was carried out in all 42 animals of the study (total of 1274 observations). We observed that in 73% of evaluations, the status found was that of closed vaginal membrane (Figure 11B), while in 13% the membrane was partially open (Figure 1C). The vaginal membrane was found to be open in 14% of observations (Figure1D).

The vaginal occlusive membrane broke at least once in 39 of the 42 females (92.85%) under evaluation. Of the total number of females with open vaginal membrane, 23 (58.97%) had a second opening, with an interval between openings from 7 to 21 days, while six females (15.38%) had a third opening, at intervals between 15 to 18 days. We observed a variation in the period during which the vaginal membrane remained open, from 1 to 12 days. The duration of the interval in between membrane openings varied between 7 and 21 days, with a higher frequency at 17 and 18 days, totalling 44.81% of the intervals observed (Table 1).

**Table 1:** Observation of opening of vaginal membrane (OVM) in *Cavia porcellus*.

**OVM**	**Females (N)**	**Average age and standard deviation (days)**	**Permanence of OVM (days)**	**Average duration and standard deviation (days)**	**Interval in betwen OVM (days)**
1º	39	35±9.75	1 – 12	3±2	-
2º	23	48±7.91	1 – 7	2±1.33	7 – 21
3º	6	53±4.88	1 - 3	2±0.81	15 - 18

### Vaginal exfoliative cytology of *Cavia porcellus*

**Figure 2:**
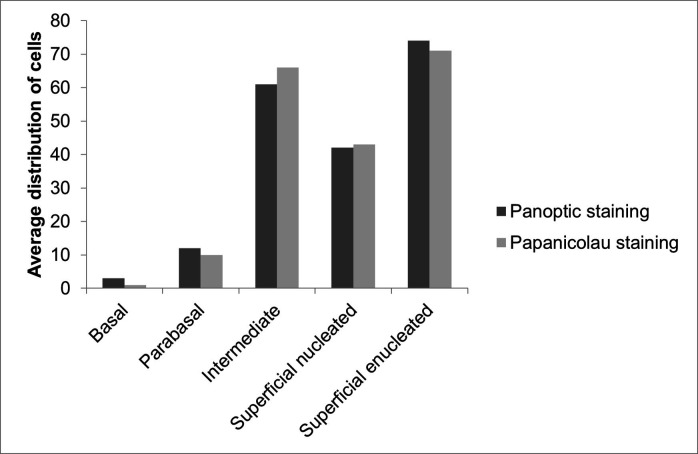
Distribution of cell types of vaginal epithelium of *Cavia porcellus* between 15 and 60 days old, classified as basal, parabasal, intermediate, superficial nucleated, and superficial enucleated.

Overall, 164 slides were prepared and evaluated, to analyze the average distribution of the cell types found. The average distribution of cell types of the vaginal epithelium shows a predominance of enucleated and intermediate superficial cells, according to the description (Figure 2).

**Figure 3:**
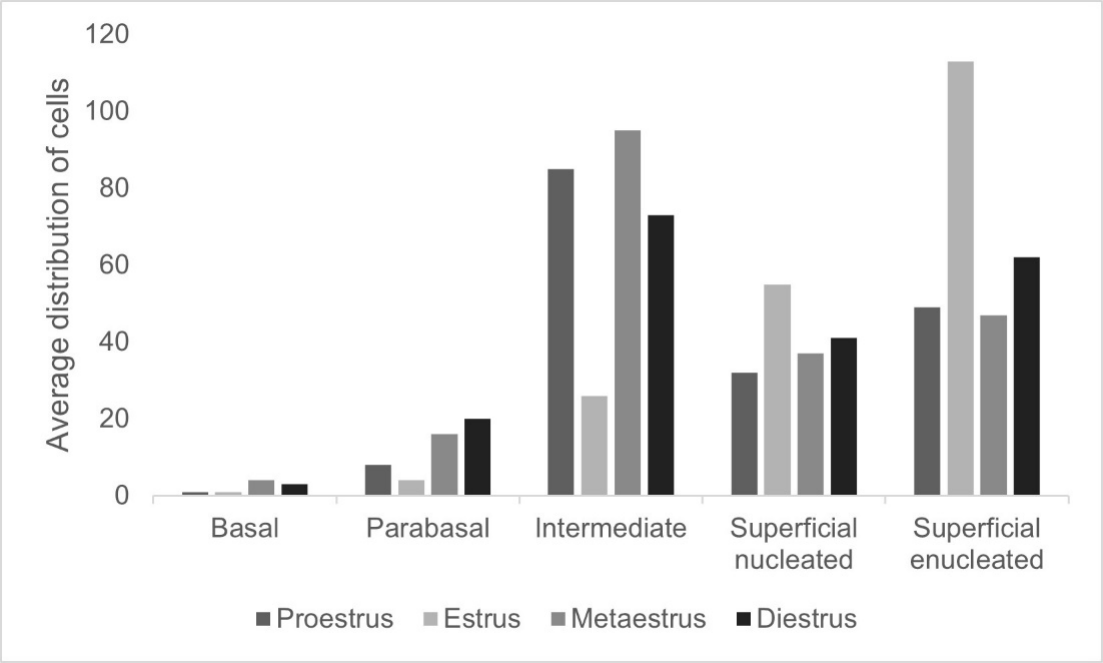
Quantitative distribution of cell types in the characterization of estrous phases in Guinea pigs (*Cavia porcellus*) kept in a laboratory animal facility.

When characterizing the phases of the estrous cycle in Guinea pigs (n = 39), we observed that during proestrus intermediate cells were in higher number (99 ± 27.3 of the total cells counted) and a light presence (+) of mucus and leukocytes. During estrus, superficial cells were predominant, mainly enucleated (113 ± 32.8), in addition to the absence of leukocytes and mucus. During metaestrus, intermediate cells were those most frequent, corresponding to about 95 ± 26 cells, with moderate presence of leukocytes (++) and no mucus. During diestrus we observed a more uniform distribution between nucleated, enucleated, and intermediate superficial cells and a very high number of leukocytes (+++). We observed an increase in the number of parabasal cells during metaestrus and diestrus, when compared with the other phases. No statistic difference was observed in the quantity of each cell type on the different phases of the estrous cycle (P < 0.05) (Figure 3).

### Puberty prior to 21 days of age

In 39 females that presented vaginal occlusive membrane opening, six were younger than 21 days (17 to 21 days) corresponding to 15.38% of females in the first opening of the membrane. For the females of this age group, the time membrane remained open varied between 1 and 5 days.

**Figure 4:**
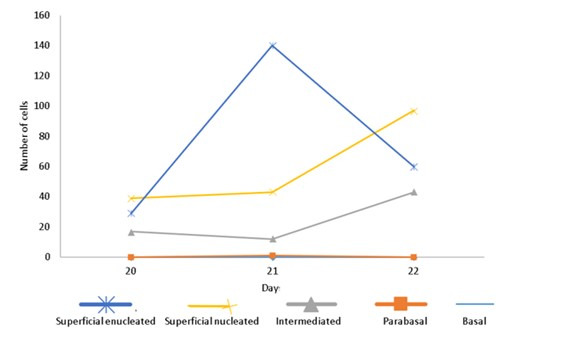
Distribution of cell types of vaginal epithelium of Guinea pig female ID 20667 in 20-22 days old.

As for the distribution of total superficial cells in vaginal smears, in animals younger than 21 days (n = 6). One 21 days old female showed a counting above 140 superficial cells (nucleated and enucleated), low presence of intermediate, parabasal and basal cells, as observed in Figure 4. In one of the samples, the material showed high concentration of mucus and formation of lumps, making it impossible to differentiate and count the cell types.

**Figure 5:**
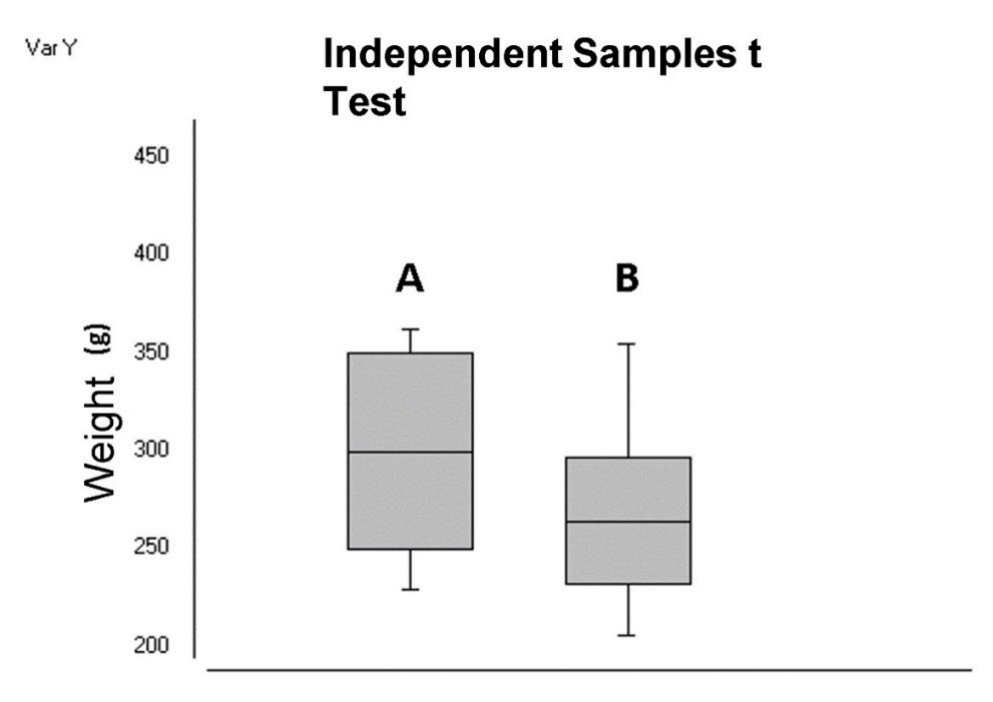
Body weight of females whose vaginal membranes opened before 21 days of age (A) and after 21 days of age (B).

The study observed a significant difference (p=0.02) between average body weight values between the six females with vaginal membrane opening before 21 days of age (299 ± 45.42 g) and the 33 females whose vaginal membrane opened at more than 21 days of age (264 ± 32.03 g) (Figure 5).

## Discussion

In this study it was possible to prove, via vaginal cytology analysis and observation of vaginal occlusive membrane, the occurrence of early puberty in *C. porcellus*. Of the 42 females under study, six had vaginal occlusive membrane opening before 21 days of age. Trillmich et al. (2006) observed that the first opening of the vaginal occlusion membrane in a group of guinea pigs under similar conditions occurs around 26 days of life. These investigators considered that the first day of opening the vaginal occlusion membrane corresponds to the beginning of the estrus phase, based on studies by Young (1937) and Touma et al. (2001). However, they did not perform serological or cytological investigations to conclude this finding in their studies.

Stockard and Papanicolaou (1919) described vaginal occlusive membrane rupture as a reliable sign of estrus. However, this condition is not the only mark of estrus. Therefore, beginning with the examination of vaginal exfoliative cytology, it was possible to characterize estrus in at least one of the females younger than 21 days, in which we observed a higher prevalence of superficial cells, a marker of this phase of the cycle in Guinea pigs (Kühnel and Mendoza, 1992; Lilley et al., 1997).

It should be highlighted that the fact that evidence of estrus was observed on the vaginal smear in only one female younger than 21 days does not rule out the possibility of occurrance in the other five females that presented early opening of the vaginal occlusive membrane. A revision of literature showed a duration of the estrous phase between 8 and 24 hours, according to Lilley et al. (1997), and between 8 and 11 hours, according to Shomer et al. (2015). The study by Alkhalaf et al. (1992) shows that the estrous phase corresponds to the second day of vaginal membrane opening, with ovulation taking place between 28 and 36 hours after opening, usually at night, between 6 p.m. and 6 a.m. (Lilley et al., 1997). Therefore, for a better following of estrus, it would be necessary to use a smaller interval in between cytological samplings, as already previously suggested by Lilley et al. (1997). However, we chose not to collect more than one sample per day, as this would mean more frequent handling of the animals and consequently higher stress levels, which may compromise the study, especially as these were very young females. We therefore chose to adopt a methodology that caused less disturbances in the daily management routine of the animals under study, to preserve their well-being and aligning with the Three Rs in animal research according to Russel and Burch (1959). In addition, unlike Lilley et al. (1997), in the present study the rupture of the vaginal occlusive membrane was not mechanically forced. All material samples for vaginal cytology in this study were collected only during natural rupture intervals, considering the fact that the study was carried out on very young females, up to 60 days old.

The significant difference observed in the average values of body weight at 20 days of age between females with vaginal occlusive membrane rupture before and after 21 days of age corroborates the study developed by Bauer et al. (2008), in which they show that animals with different weight gain were younger when their first estrous cycle took place.

The results, in the conditions reported herein, showed a wide range in the age of spontaneously opening of vaginal occlusive membrane, between 15 and 60 days old.

Trillmich et al. (2006) suggest that the presence of the mother and the breeding male that present a courtship vocalization behavior can induce the precocity of estrus in young guinea pigs, as they observed in their studies that, in the family groups of guinea pigs kept the males of the family together with the young females, until what they consider as the 1st estrus, there was a complete opening of the vaginal membrane around 26 days. However, they could not clearly distinguish the effect or confirm it as an inducer of the opening of the vaginal membrane in young females.

The time during which the vaginal membrane remained opened for the first time varied greatly (1-12 successive days) and was higher than that reported by other authors (Alkhalaf et al., 1992; Luna et al., 2003), who described a period of membrane opening closer to what was observed during the second opening verified in the current study (1-7 days). It should be emphasized, however, that only two females had opening periods higher than that described by other authors (8 and 12 days). In addition, the higher frequency of membrane opening period (between 1 and 3 days) corresponded to the study by Araníbar and Echevarría (2014). Macroscopic observation of the vulva showed morphological differences, such as swelling, redness and secretion, like those described by Li and Shen (2015).

The duration of the interval in openings showed a variation between 7 and 21 days, occurring with a higher frequency to 17 and 18 days, higher than those presented in literature, which describe cycles between 15 and 17 days (Lilley et al., 1997; Stockard and Papanicolaou, 1919; Hubrecht, 2010), or between 14 and 16 days (Kühnel and Mendoza, 1992; Wang et al., 2010; Li and Shen, 2015).

Vaginal cytology proved to be reliable for the characterization of the estrous cycle. There was distribution of cell types of the vaginal epithelium in each phase, as in the study by Li and Shen (2015). During proestrus, intermediary cells were observed in a higher number, with moderate presence of mucus. This aspect was also considered a characteristic of the proestrus phase by Kühnel and Mendoza (1992). During estrous, the predominant cells were superficial ones, mostly enucleateed, while Kühnel and Mendoza (1992) and Lilley et al. (1997) observed a more effective presence of nucleated superficial cells in this phase. During metoestrous and diestrous, cell types observed were like those described in the work of Kühnel and Mendoza (1992), with a high number of leukocytes and increased number of parabasal cells. For differentiation, the slides characterized as diestrous were those collected from the fifth day of vaginal membrane opening on, period that Alkhalaf et al. (1992) and Shomer et al. (2015) consider the beginning of this phase.

Thus, observation of the rupture of vaginal occlusive membrane, together with the characterization and incidence of cell types through vaginal cytology, made it possible to detect estrous in Guinea pigs, supporting previous reports in studies by Li and Shen (2015).

This study provides important and novel information on the reproductive biology of young *C. porcellus* females with the evidence of early estrous (before 21 days) and the characterization of at least three estrous cycles before 60 days old.

## Conclusion

The Guinea pig females kept in laboratory animal facilities conditions showed that the rupture of the occlusive membrane occurs gradually, in some cases, with a partially open phase that makes cytological sampling impossible. Differences in body development (weight) of the females with early puberty is one of the possible factors in the distribution of age in the opening of the vaginal membrane.

In the conditions in which the study was carried out, Guinea pigs showed vaginal occlusive membrane rupture and vaginal cytology characteristic of estrous at an age below 21 days, pointing to puberty before the standard weaning period. Because of this observation, in laboratory animal facilities is necessary to modify the Guinea pigs management practices aiming to adapt the separation of female daughters from their fathers at 16 days old.
